# Natural history of postnatal rhesus cytomegalovirus shedding by dams and acquisition by infant rhesus monkeys

**DOI:** 10.1371/journal.pone.0206330

**Published:** 2018-10-24

**Authors:** Amitinder Kaur, Hannah L. Itell, E. Peek Ehlinger, Valerie Varner, Soren Gantt, Sallie R. Permar

**Affiliations:** 1 Tulane National Primate Research Center, Covington, Louisiana, United States of America; 2 Molecular and Cellular Biology PhD Program, University of Washington, Seattle, Washington, United States of America; 3 Alaska Family Medicine Residency, Anchorage, Alaska, United States of America; 4 Center for Virology and Vaccine Research, Beth Israel Deaconess Medical Center/Harvard Medical School, Boston, Massachusetts, United States of America; 5 BC Children’s Hospital Research Institute, University of British Columbia, Vancouver, British Columbia, Canada; 6 Human Vaccine Institute, Duke University Medical Center, Durham, North Carolina, United States of America; Texas Woman’s University, UNITED STATES

## Abstract

**Background:**

Human infants frequently acquire human cytomegalovirus (HCMV) through breastfeeding, resulting in persistent high-level viral shedding in saliva and urine and infectivity to others, including pregnant women. Thus, vaccination to interrupt postnatal HCMV transmission is an attractive strategy to prevent HCMV spread and congenital infection. Rhesus CMV (RhCMV) in nonhuman primates is a valuable model for the study of immune strategies to prevent CMV transmission. Although rhesus monkeys typically acquire RhCMV before 1 year of age, the timing and mode of natural infant RhCMV transmission remain unknown.

**Methods:**

We followed 5 RhCMV-seropositive dams and their infants from birth until weaning, approximately 6 months later. RhCMV DNA levels in plasma, breast milk, saliva, and urine were measured every 2 weeks by quantitative PCR. RhCMV-specific T cell responses in peripheral blood and breast milk were measured by interferon gamma ELISpot assays. Serum IgG antibody levels were measured by ELISA.

**Results:**

Four of five postpartum RhCMV-seropositive mothers had intermittent, low-level RhCMV shedding in breast milk, whereas all had high-magnitude RhCMV shedding in saliva and urine. The kinetics of maternal blood RhCMV-specific T cell responses and viral shedding in urine and saliva did not strongly associate, though dams with consistently high systemic RhCMV-specific T cell responses tended to have undetectable RhCMV shedding in breast milk. All RhCMV-exposed infants had intermittent, low-level RhCMV shedding in saliva during the lactation period, with minimal systemic RhCMV-specific T cell responses.

**Conclusions:**

Despite exposure to RhCMV shedding in breast milk and other maternal fluids, postnatal mother-to-child RhCMV transmission appears to be less efficient than that of HCMV. A greater understanding of the determinants of RhCMV transmission and its usefulness as a model of HCMV mucosal acquisition may provide insight into strategies to prevent HCMV infections in humans.

## Introduction

Human cytomegalovirus (HCMV) is a ubiquitous human virus, infecting more than half of the U.S. population [1] and >90% of populations in developing regions [2]. HCMV is primarily transmitted through mucosal fluids, including saliva, genital fluids, and breast milk. It is the most common congenital infection worldwide and a leading cause of mortality in individuals undergoing transplantation. HCMV-infected infants and young children persistently shed high levels of virus in saliva and urine and constitute an important source of HCMV transmission to other individuals, including pregnant women [3–10]. Breastfeeding is a major route of postnatal HCMV transmission to infants [11, 12]. Importantly, preterm infants who acquire HCMV via breast milk can develop a sepsis-like illness, complicating the optimal nutrition strategies for these highly vulnerable infants [13]. Thus, a vaccine interrupting postnatal HCMV transmission to infants could be a practical strategy for limiting viral spread to pregnant women with enormous potential to reduce congenital infection and disease [14]. The development of a preclinical animal model of postnatal CMV acquisition would expedite the development of an effective HCMV vaccine.

The strict species-specific tropism of CMV precludes the direct study of vaccine approaches for HCMV in animal models. Small animal models have been developed to study immune protection against species-specific CMV, with the guinea pig model being widely used for studying congenital CMV transmission [15–17]. However, this model lacks anatomic and physiologic similarities to human pregnancy. Moreover, many of the genetic differences of guinea pig CMV and HCMV are yet to be uncovered [18]. On the other hand, primate CMV strains have considerable genetic and functional homology to that of HCMV, with the best studied being rhesus CMV (RhCMV) [19–21]. Intrauterine inoculation of RhCMV leads to fetal pathology similar to that of congenital HCMV infection [22]. We recently established a novel congenital CMV infection model in rhesus macaques and showed that RhCMV can cross the placenta of RhCMV-naïve dams following experimental infection in the first trimester of pregnancy [23, 24]. Furthermore, oral RhCMV inoculation of infant and adult rhesus monkeys can establish chronic infection [25]. Thus, in addition to congenital infection, RhCMV may represent a useful model of postnatal transmission to inform the development of an effective vaccine to prevent postnatal HCMV infection.

Although the overwhelming majority of members of rhesus monkey colonies in the wild [26] and in captivity [27] are RhCMV-seropositive, the natural history of virus acquisition remains unclear. Similar to that of HCMV infection [12, 28], primary RhCMV infection of rhesus monkeys is asymptomatic and results in chronic shedding of virus in saliva and urine [29–31]. Infant rhesus monkeys typically become IgG seropositive by one year of age [32], but it is unclear if the virus is acquired from their mother, e.g. via breast milk, or from other colony members. Although HCMV-seropositive women frequently shed HCMV in breast milk, it is unknown whether breast milk shedding of RhCMV is similar. Determining the magnitude and kinetics of postpartum RhCMV shedding and the typical mode of infant RhCMV acquisition is central to establishing a model of postnatal RhCMV transmission in rhesus monkeys. We therefore conducted a longitudinal study to characterize the natural history of maternal RhCMV shedding and transmission to infant rhesus monkeys.

## Materials and methods

### Animals and sample collection

Five RhCMV-seropositive, pair-housed rhesus macaque mothers and their infants originating from the specific pathogen free (SPF) colony at the New England Primate Research Center (NEPRC) were followed for the development of RhCMV viremia, mucosal RhCMV shedding, and RhCMV-specific immune responses from parturition until the end of the weaning period, approximately six months (26 weeks) following delivery. One of the RhCMV-seropositive mothers was inoculated and productively infected with SIVmac251 during early pregnancy (monkey 196) as part of a prior study. An additional mother-infant pair from the herpesvirus-free “superclean” SPF colony was included in the study as a RhCMV-seronegative control. Maternal blood, urine (pan-collected), saliva (collected via swab placed into 1 mL of PBS), and breast milk (collected via manual breast massage/stripping) and infant saliva (collected via swab placed into 1 mL of PBS) and blood were collected at the first available time point postpartum (2–10 weeks) and every two weeks until weaning.

The institutional animal care and use committee at Harvard Medical School approved all procedures in this observational study (Protocol #04772). Pair-housed dams and pre-weaned infants participated in the NEPRC Environmental Enrichment program before infants were weaned and introduced to social housing. To minimize animal suffering, oral swabs and oral washes were employed in the place of more invasive methods such as biopsies. Likewise, instead of catheterization, urine was collected from a pan. To ensure animal distress was minimized, veterinary staff observed the animals twice daily for signs of abnormal behavior. For breast milk, saliva, and blood collections, dams and weaned infants were sedated with 10–15 mg/kg of ketamine HCl or 4–10 mg/kg of telazol intramuscularly, while pre-weaned infants were manually restrained. Blood collections from dams (up to 10 mL) and infants (up to 1 mL) were obtained from peripheral veins, commonly the femoral vein.

### Quantitation of RhCMV virus load

DNA was extracted from urine, saliva, breast milk, and plasma samples using QIAGEN DNA extraction columns. RhCMV virus load was measured by quantitative PCR (qPCR) with primers and probe specific for exon 1 of the immediate early (IE) gene, as previously described [33]. Three replicates were initially performed on each sample type. If only one of the triplicates had a detectable result, an additional three replicates were performed. For infant saliva and plasma, nine replicates were routinely performed. With this replicate schema, a sample was only considered positive if RhCMV was detected in two or more replicates. Viral load for positive samples is reported as the median and range of the two or more positive qPCR replicates.

### Measurement of peripheral blood mononuclear cell (PBMC) and breast milk cell immunodominant RhCMV-specific cellular responses

In accordance to a previously described SIV-specific ELISpot assay, adapted here to measure RhCMV-specific responses [34], PBMCs (2 x10^5^) were incubated overnight with overlapping peptide pools of the IL10, IE1, IE2, and/or pp65 homolog RhCMV gene products in wells coated with anti-IFNγ monoclonal antibody (mAb). IFNγ release was measured by alkaline phosphatase-labeled anti-IFNγ polyclonal Ab and substrate-induced spot formation. Maternal PBMCs were assayed in triplicates, but, due to low cell numbers, infant PBMCs did not have assay replicates. The peptide pool that elicited the highest magnitude peripheral blood response in each maternal subject at the initial time point was selected as the immunodominant response and was measured over time in each monkey. Breast milk cells (2 x10^4^) were incubated with 1x10^5^ mitomycin-C-treated autologous PBMC and the immunodominant RhCMV gene product peptides in anti-IFNγ mAb-coated wells of a 48-well plate for 6 days prior to testing for IFNγ release in an ELISpot assay. As with the infant PBMCs, ELISpot assays for breast milk cells were not run in replicates due to low cell number. The cut-off for the assay is SFU at least twice the background and >50 SFU/10^6^ cells. Additionally, breast milk cell data was only reported for each time point when the positive control, the superantigen *Staphylococcus aureus* Enterotoxin Type B (SEB), had a response greater than twice the background to demonstrate that adequate cell numbers were present to detect a response. ELISpot responses are reported in spot forming units (SFU) per 10^6^ cells, and the median and range of triplicate wells is reported for maternal PBMC results. The overall associations between the kinetics of RhCMV-specific cellular responses and viral shedding across compartments were evaluated by Spearman Rank Tests, by combining longitudinal data from all five dams or infants.

### Measurement of RhCMV-specific IgG response

RhCMV-specific IgG was measured in infant plasma by a previously described whole virion ELISA, which uses a 96-well plate coated with 880 PFU/mL of filtered, fibroblast-passaged RhCMV strain 180.92 [23, 35]. Plasma (1:20 dilution) was incubated in duplicate wells and RhCMV-binding IgG was detected using an HRP-conjugated goat anti-monkey IgG Ab (sc-2458, Santa Cruz Biotechnology) and substrate incubation. The magnitude of the RhCMV-specific IgG binding responses is reported as optical density (OD) at 450 nm.

## Results

### Magnitude and kinetics of postpartum maternal RhCMV shedding

**Fig 1:**
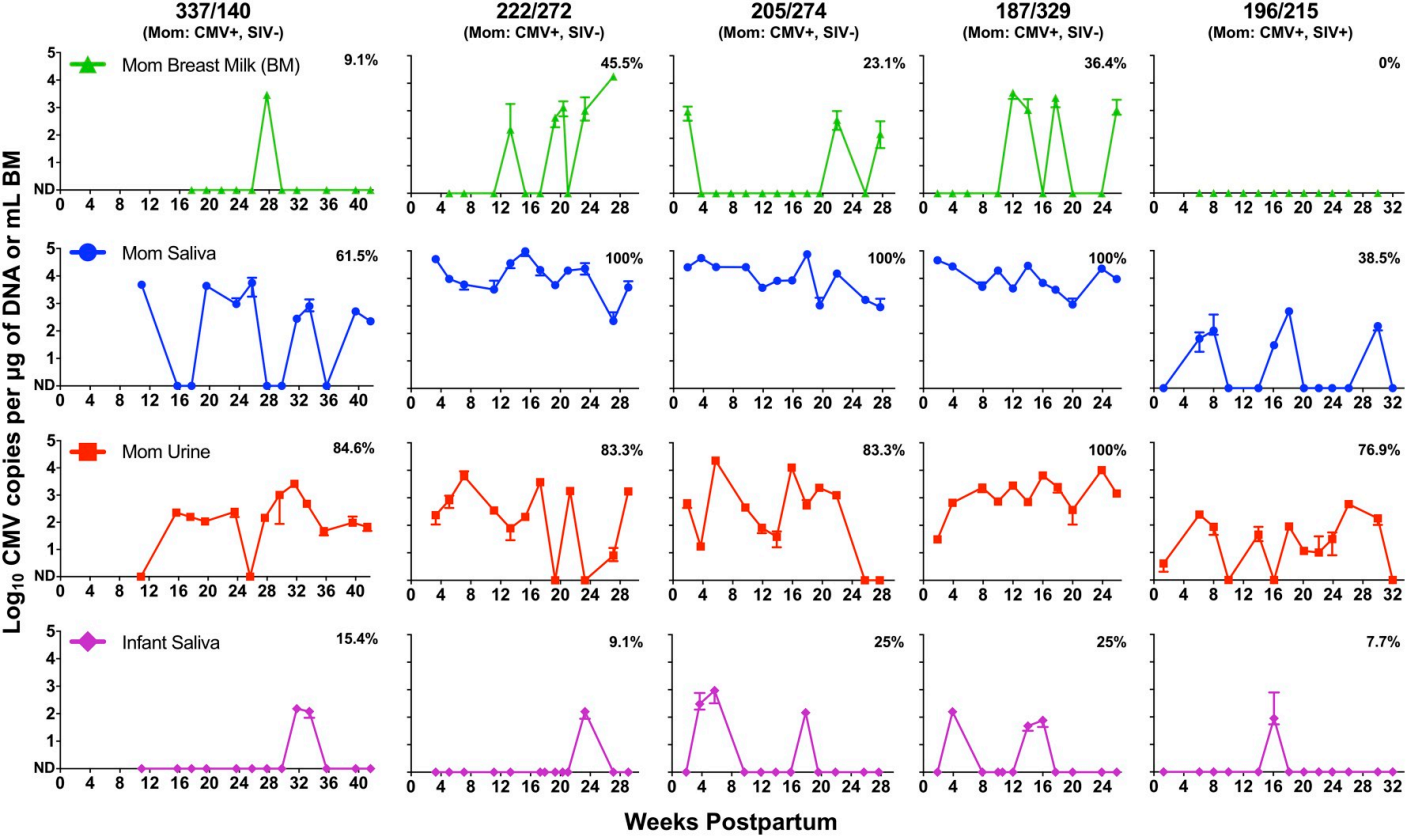
The frequency and magnitude of postpartum RhCMV shedding in mothers and infants. Maternal breast milk, saliva, and urine and infant saliva were routinely assessed for RhCMV shedding by qPCR during the lactation period, until approximately 26 weeks postpartum, and for follow-up time points. The median and range of the positive qPCR replicates for each time point and sample type is reported as CMV DNA copies per μg of DNA for saliva and urine and as CMV DNA copies per mL of breast milk (BM). Samples with less than two positive qPCR replicates were considered not detectable (ND). The frequency of detectable samples across the assessed time points for each animal and sample type is denoted on each plot.

To characterize the postnatal exposure of infants to RhCMV, we measured maternal RhCMV shedding in urine, saliva, and breast milk over time. Not surprisingly, RhCMV shedding was common in maternal saliva and urine, as all RhCMV-seropositive mothers had detectable RhCMV shedding in these mucosal fluids with an overall detection frequency of 38.5–100% during the assessed postpartum period (Fig 1). Three of the RhCMV-seropositive mothers had particularly high-magnitude RhCMV shedding in urine and saliva during this period (Fig 1; monkeys 222, 205, 187; median copy number of detectable time points: 14,478, 11,727, and 9,467 copies/μg of DNA in saliva and 584, 581, and 1,431 copies/μg of DNA in urine), with consistent detection (100% in saliva, 83.3–100% in urine) across all time points. In the other two RhCMV-seropositive mothers (monkey 337 and the SIV/RhCMV co-infected monkey 196), saliva and urine RhCMV shedding was low-level and intermittent (median copy number of detectable time points: 158 and 66 copies/μg of DNA in saliva and 882 and 152 copies/μg of DNA in urine). In these monkeys, RhCMV shedding during the assessed postpartum period was more frequently detected in urine (76.9–84.6%) than in saliva (38.5–61.5%). Consistent with the viral kinetics of chronic RhCMV infection, no virus was detected in the plasma of any of the RhCMV-seropositive mothers. As expected, RhCMV DNA was not detected at any sampled postpartum time point in the maternal or infant saliva, or maternal plasma and urine, of the one control RhCMV-seronegative mother-infant pair.

Similar to the pattern observed in HCMV-seropositive women [36–40], RhCMV shedding in breast milk was intermittently detected in four of five RhCMV-seropositive monkeys (Fig 1). RhCMV shedding in breast milk was detected less frequently and with more variable magnitude than that in urine and saliva (0–45.5% detection in milk over the assessed postpartum time points). However, the magnitude of the RhCMV shedding detected in milk (monkeys 337, 222, 205, 187; median copy number of detectable time points: 2,886, 1,007, 464, and 2,025 copies/mL; range: 142–17,781 copies/mL) is similar to previously reported levels of HCMV shedding in human milk [41], albeit these assays would have distinct sensitivities based on the different primer pairs required to detect HCMV versus RhCMV. Interestingly, the only mother without detectable RhCMV in breast milk was the SIV and RhCMV-coinfected mother, monkey 196. RhCMV shedding was also not detected in breast milk of the RhCMV-seronegative control dam.

### Maternal RhCMV-specific cellular immune responses and RhCMV shedding

**Fig 2:**
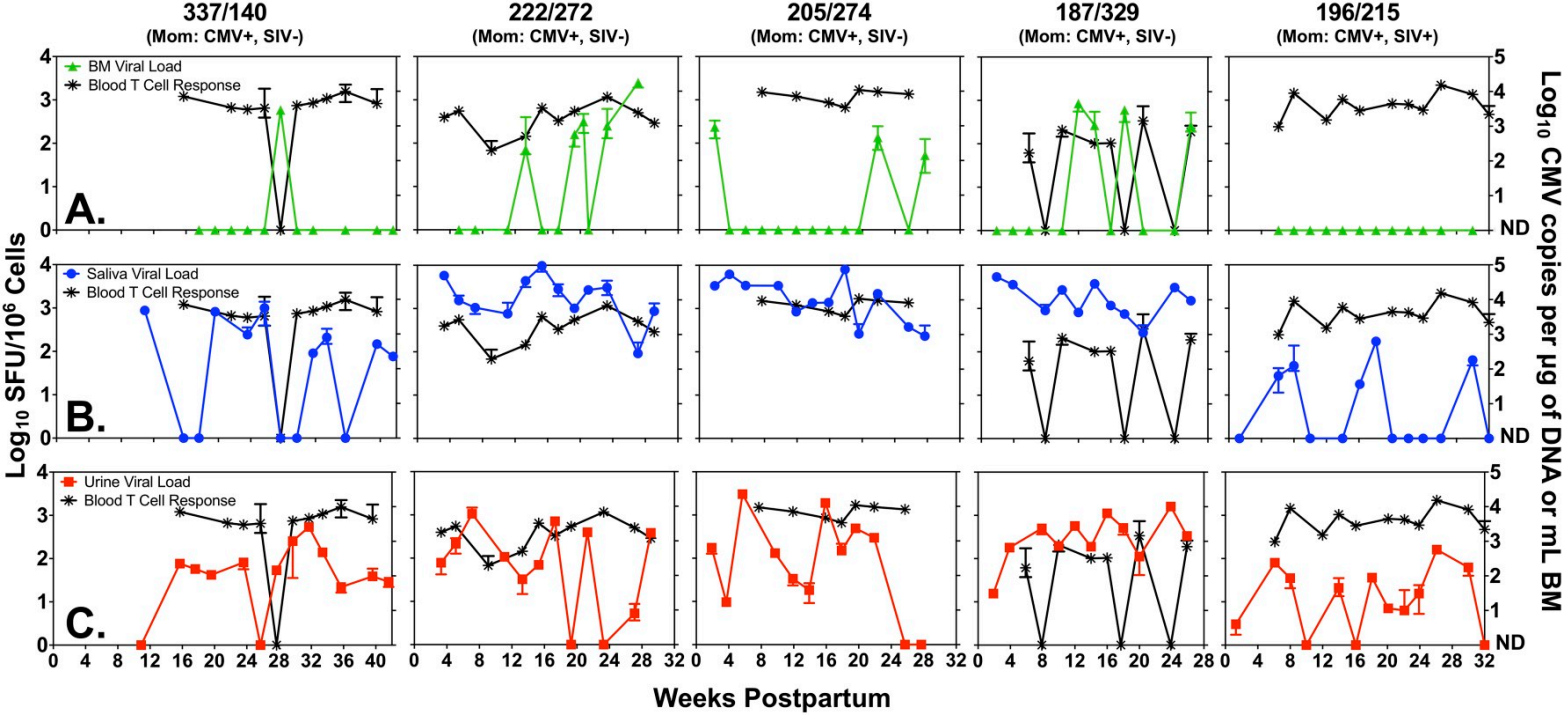
The kinetics of maternal systemic T cell responses and RhCMV shedding. Maternal systemic T cell responses were measured by IFNγ ELISpot, using the immunodominant peptide pool for each dam (monkeys 337 and 222: pp65; monkeys 196 and 187: IE-1; monkey 205: IE-2). RhCMV virus load was measured by qPCR in maternal breast milk (**A**, reported as copies/mL) and in maternal saliva and urine (**B-C**, both reported as copies/μg of DNA). The median and range of the positive qPCR replicates are indicated when 2 or more replicates were positive.

We next assessed the association of maternal RhCMV-specific systemic cellular immune responses with the viral load of RhCMV shedding in mucosal secretions. We first screened each RhCMV-seropositive mother to determine the immunodominant RhCMV-specific immune response in peripheral blood, as detected by PBMC IFNγ ELISpot against the gene products of the RhCMV homologs of pp65, IE-1, IE-2, and IL-10. The immunodominant response was defined as the RhCMV gene product peptide pool that elicited the strongest virus-specific IFNγ response in an overnight peptide-stimulation ELISpot assay at a prepartum time point. The selected immunodominant peptide pool (monkeys 337 and 222: pp65; monkeys 196 and 187: IE-1; monkey 205: IE-2) was then used to determine the kinetics of RhCMV-specific cellular responses in blood throughout the postpartum period (Fig 2A and 2C). The immunodominant RhCMV-specific cellular response was consistently detected in the blood in the majority of time points for four of five mothers, and intermittently detected at a lower magnitude in monkey 187. The kinetics of the immunodominant RhCMV-specific immune response were then compared to the kinetics of virus shedding in the milk, saliva, and urine (Fig 2A and 2C). The kinetics of the blood RhCMV-specific cellular responses did not show a consistent temporal association with the kinetics of virus shedding in urine and saliva (Fig 2B and 2C). However, during periods of consistent, high RhCMV-specific cellular responses, RhCMV shedding in breast milk was typically undetectable (monkeys 337, 205, and 196). Despite these observations, the kinetics of maternal systemic immunodominant T cell responses were not correlated with the kinetics of viral shedding in breast milk (Spearman Rank Test; r = -0.21, p = 0.17) nor urine (r = -0.20, p = 0.20) and only weakly correlated with oral viral load over time (r = -0.34, p = 0.03).

**Fig 3:**
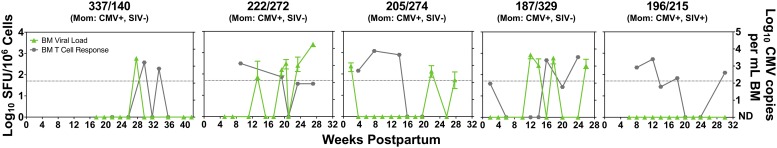
The kinetics of RhCMV-specific T cell responses and RhCMV viral load in breast milk. Maternal RhCMV-specific immunodominant T cell responses (IE1, IE2, or pp65) in breast milk were measured by cultured IFNγ ELISpot (grey line), using the immunodominant peptide pool for each dam (monkeys 337 and 222: pp65; monkeys 196 and 187: IE-1; monkey 205: IE-2). Breast milk virus load (green line) was measured by qPCR, and the median and range of the positive qPCR replicates are indicated when 2 or more replicates were positive. T cell responses were considered positive if they were greater than twice the background and 50 SFU/10^6^ cells (dotted line).

As breast milk is known to be a source of CMV-specific T cell responses [41, 42], we assessed the magnitude and kinetics of RhCMV-specific cellular responses in breast milk in relation to RhCMV shedding in the same compartment. However, due to the low number of cells able to be isolated from rhesus milk samples, we were unable to detect T cell IFNγ secretion in response to overnight incubation with either RhCMV peptides or our positive control, the superantigen SEB. Thus, we developed a cultured ELISpot assay in which breast milk cells are incubated with mitomycin C-treated autologous PBMCs pulsed with the immunodominant peptide pool defined for each monkey (see Materials and methods) for one week prior to detection of breast milk cell IFNγ secretion. The assay was only considered valid if a response was detected against SEB at the same time point. Using this method, we were able to detect RhCMV-specific cellular responses in milk of all five RhCMV-seropositive mothers (Fig 3). Strong milk RhCMV-specific cellular responses were detected both in association with a spike of virus replication in milk and during periods without detectable virus shedding, and the overall correlation between the longitudinal data of these two components were not significantly correlated (Spearman Rank Test, r = -0.25, p = 0.21). Thus, there was not a relationship between RhCMV shedding and detection of cellular responses in breast milk of rhesus monkeys, consistent with the lack of association between these parameters in human milk [41].

### Virologic and immunologic assessment of infant RhCMV acquisition during lactation

We assessed the RhCMV-exposed infants for evidence of primary RhCMV infection using RhCMV qPCR of plasma and saliva. Importantly, we did not detect RhCMV in the first postpartum plasma or saliva sample available from any infant monkey, suggesting that there was no *in utero* virus transmission (Fig 1). We did detect RhCMV in plasma in only one RhCMV-exposed infant (infant monkey 274) at a single time point 25 weeks after birth (144 copies/mL). Additionally, all five infants of RhCMV-seropositive mothers had infrequent, low copy number RhCMV shedding in saliva during the period of breastfeeding (Fig 1; 7.7–25% detection). Notably, three of these five monkeys (infant monkeys 140, 274, and 329) had two consecutive saliva samples positive by qPCR, which may indicate oral virus replication [43], albeit of a transient nature because subsequent sampling time points were negative.

**Fig 4:**
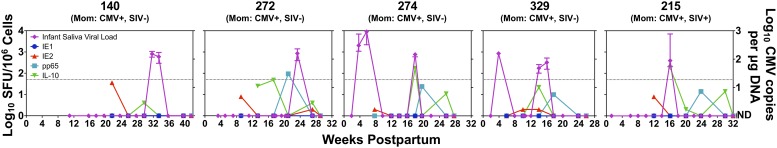
Infant RhCMV-specific T cell responses and virus shedding. RhCMV-specific T cell responses against IE1, IE2, pp65, and IL-10 RhCMV homologs were measured in peripheral blood of each infant monkey by IFNγ ELISpot. A response was considered positive if it was greater than twice the background and greater than 50 SFU/10^6^ cells (dotted line). Infant saliva viral load was measured by qPCR and is plotted as the median and range of the positive qPCR replicates.

In addition to infant RhCMV shedding, we also assessed for infant RhCMV-specific cellular and humoral immune responses during postpartum period. We measured infant cellular immune responses every two weeks during breast milk RhCMV exposure using an overnight peptide-stimulation IFNγ ELISpot against the RhCMV homologs of IE-1, IE-2, pp65, and IL-10 (Fig 4). Responses were considered positive by having twice the number of spots in peptide-stimulation wells compared to the media-only wells and greater than 50 spots/10^6^ PBMCs. Three infants had positive RhCMV-specific cellular immune responses detected against a single peptide pool at a single time point (infant monkey 272: pp65, 21 weeks old; infant monkey 274: IL-10, 18 weeks old; infant monkey 215: IL-10, 16 weeks old). Notably, detection of an IFNγ response against the RhCMV IL-10 peptide pool was concurrent with detection of RhCMV in the saliva of infant monkeys 274 and 215, though the timing of the weak RhCMV pp65-specific cellular response in monkey 272 was distinct from the detection of RhCMV in saliva (Fig 4). Due to there only being three instances of infant systemic cellular responses exceeding the assay cutoff, we did not have sufficient data to correlate these immune responses to infant oral viral load. Thus, in contrast to the strong and consistent ELISpot responses observed in infected dams, RhCMV-specific cellular responses in infants were infrequent and low-level, even in the three infants with evidence of transient oral RhCMV replication.

**Fig 5:**
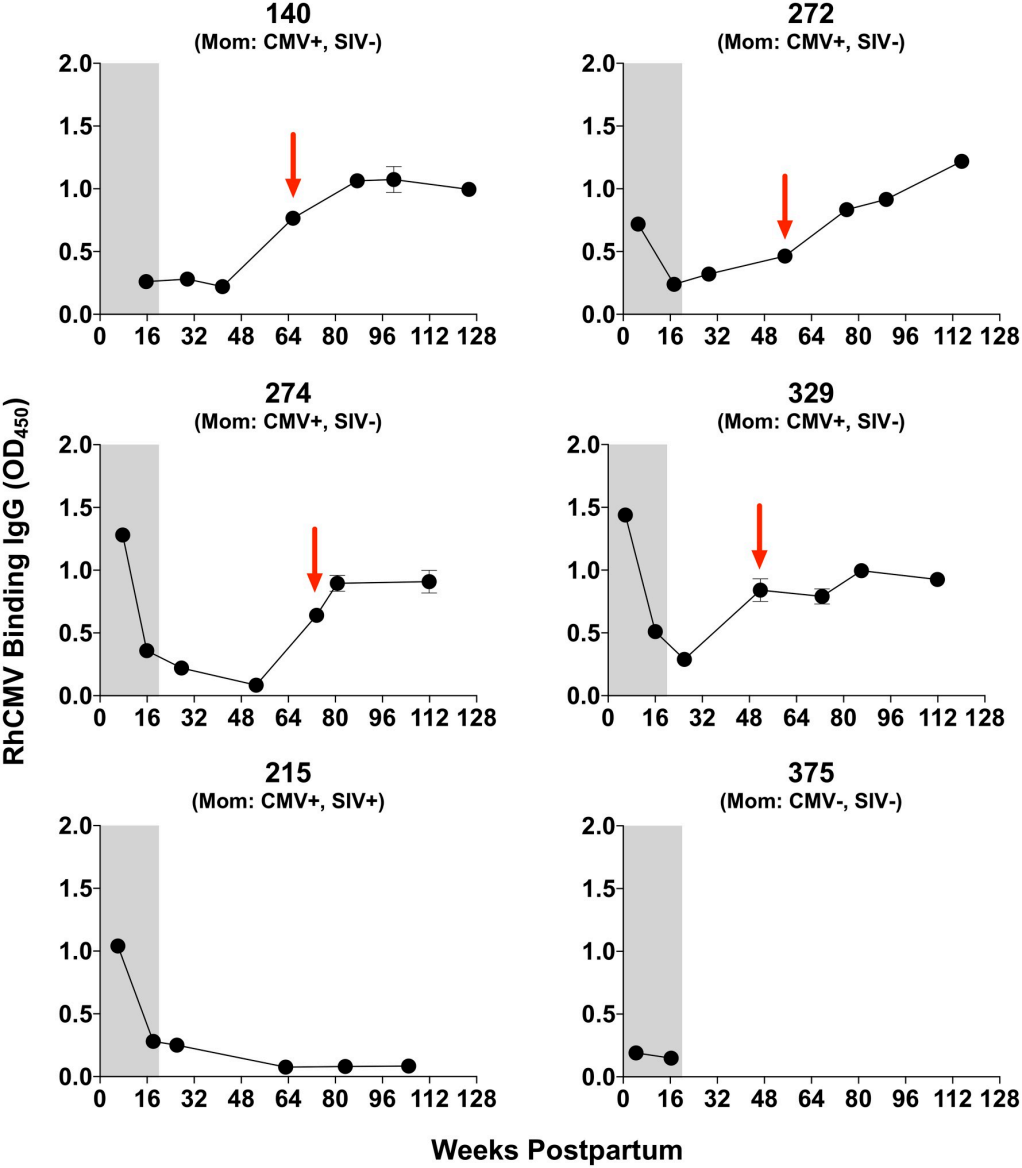
Infant RhCMV IgG responses during and after the period of lactation. Infant RhCMV-binding IgG responses were measured by a whole virion RhCMV ELISA and are plotted as the mean and error of technical duplicates. Infant antibody responses through the 16-week postpartum time point (shaded region) represent congenitally-acquired maternal antibody. Infants were weaned and introduced to group housing approximately 26 weeks postpartum. Red arrows indicate infant seroconversion after introduction to group housing.

Finally, we assessed the kinetics of RhCMV-specific IgG responses in infants during and after the period of breastfeeding. As expected, infants of RhCMV-seropositive mothers had strong RhCMV-specific IgG responses detected at birth (Fig 5), consistent with placentally-acquired maternal antibody. By 32 weeks postpartum, this passively-acquired maternal antibody waned to levels similar to that of the RhCMV-unexposed infant (infant monkey 375). Subsequently, four of the five RhCMV-exposed infants demonstrated a rise in RhCMV-specific IgG between 48 and 80 weeks postpartum, indicative of seroconversion. Importantly, by the time of these seroconversion events, the infants had already been weaned and introduced to group housing. Remarkably, infant 215 did not seroconvert prior to two years of age, despite group housing post-weaning.

## Discussion

The availability of a nonhuman primate model of postnatal CMV transmission would greatly enhance our ability to determine the protective effect of maternal and/or infant immunologic interventions to prevent the common occurrence of infant CMV acquisition, which could inform human clinical trials of adolescents or adults evaluating new CMV vaccine candidates. While RhCMV is genetically similar to HCMV [19, 44] and is known to induce disease in fetal rhesus monkeys similar to that of congenital HCMV infection [22], the natural history of mother-to-child RhCMV transmission remains incomplete. While it has been established that infant monkeys typically seroconvert during the first year of life, it was previously unknown if infant rhesus monkeys, like human infants, are exposed to the virus via breastfeeding. Moreover, the typical mode of infant RhCMV acquisition has not been established. Thus, we performed this observational, longitudinal study of postpartum RhCMV shedding and virus-specific immune responses in pair-housed RhCMV-seropositive female rhesus monkeys and their RhCMV-exposed infants.

In RhCMV-seropositive mothers, postpartum virus shedding was common in urine and saliva, without any evidence of viremia, consistent with chronic RhCMV infection [29, 45]. RhCMV was also shed in breast milk, though shedding was less frequently detected in milk than the other mucosal fluids. Notably, however, the magnitude of the breast milk virus shedding seems to be similar to that observed in humans [41, 46–48], though future studies will need to standardize the human and rhesus assays in order to make quantitative comparisons between viral loads. Unexpectedly, the SIV-infected RhCMV-seropositive mother displayed the least amount of virus shedding and had no detectable RhCMV in breast milk. With impairment of T cell responses associated with SIV infection and the frequently-observed opportunistic infections due to uncontrolled RhCMV and HCMV replication in infected monkeys and HIV-infected humans, respectively [49–53], we predicted that this mother would have high levels of virus shedding. However, in this mother, recent SIV infection during pregnancy did not appear to impair RhCMV-specific T cell responses (Fig 2) or result in high levels of RhCMV shedding, though the implications of these findings are limited due to our small sample size of only one RhCMV-SIV coinfected mother-infant pair. Importantly, because samples were assayed by qPCR for the presence of RhCMV DNA rather than being cultured for infectious virus, it may be possible that DNA viral load overestimated the amount of infectious virions in a given sample. Nevertheless, this potential overestimation does not undermine the trends in our findings, such as a lower magnitude of RhCMV in breast milk compared to that in saliva and urine and no breast milk shedding of RhCMV in the SIV-infected RhCMV-seropositive mother.

Interestingly, the presence of detectable breast milk RhCMV virus shedding tracked with the magnitude of the maternal systemic immunodominant RhCMV-specific cellular immune responses in some animals, whereas other animals had intermittent shedding that was not temporally associated with systemic virus-specific T cell responses. Moreover, RhCMV-specific cellular responses were detectable in the breast milk compartment yet did not appear to be associated with RhCMV shedding in breast milk, indicating that breast milk viral replication may not be controlled by T cells in the milk. In a cross-sectional human study of HCMV-seropositive women with preterm infants, we were also unable to detect a correlation between the magnitude of the HCMV pp65-specific T cell response in blood or milk and the breast milk virus load [41]. However, the cross-sectional study design and lack of selection of the immunodominant HCMV-specific response in each subject may have limited our ability to detect an association between these parameters.

Breastfeeding is a common mode of HCMV transmission [11, 12, 46], though transmission also occurs through other exposures in early childhood, such as interactions in a daycare setting [3, 4]. Although RhCMV was detected during the sampling period in breast milk of 4 of 5 RhCMV-seropositive mothers, and in saliva and urine of all, none of their infants demonstrated the persistent high-level RhCMV shedding that has been characterized in infant rhesus monkeys orally inoculated with RhCMV [25] and in human infants postnatally infected with HCMV [28, 54]. Rather, RhCMV shedding detected in saliva during early infancy was intermittent and low-level (Fig 1), and seroconversion only occurred several weeks later after introduction to group housing, which was not surprising due to the documented efficient nature of horizontal transmission among rhesus colonies [55, 56].

The transient detection of virus in infant saliva and RhCMV-specific cellular responses in infant blood during the lactation period suggests that the infrequent oral shedding observed in these infants did not represent established RhCMV infection. This pattern is consistent with transient oral HCMV infection that has been described in seronegative infants [43], though contamination of saliva by maternal breast milk or another source cannot be excluded in these animals. Additionally, our findings are not conclusive as to whether passively acquired maternal antibody protected the infants from viral acquisition during the lactation period, as a number of instances of detectable oral viral shedding in infants temporally correlated with high levels of systemic infant RhCMV-specific IgG, and vice-versa. Therefore, it is more likely that the reduced number of postnatal RhCMV transmission cases during the lactation period, compared to that typical of HCMV [11], may be due to the intermittent nature of the shedding of virus in breast milk in chronically RhCMV-infected rhesus monkey dams.

Our conclusions are limited by the small number of mother-infant pairs studied, but suggest that SIV coinfection may not increase risk of postnatal RhCMV transmission, and that breast milk transmission of RhCMV by infant monkeys may be even less efficient than that of HCMV, which appears to require multiple exposures before infection is successfully established [43]. Therefore, while this model may not be well suited for testing candidate vaccines for the prevention of breastfeeding transmission, the rhesus monkey model may prove valuable for identifying the viral and/or host immune determinants of postnatal HCMV transmission, which could guide the development of a vaccine to prevent HCMV mucosal transmission.
